# When simplicity triumphs: niche specialization of gut bacteria exists even for simple fiber structures

**DOI:** 10.1093/ismeco/ycae037

**Published:** 2024-04-11

**Authors:** Haidi Xu, Nicholas A Pudlo, Thaisa M Cantu-Jungles, Yunus E Tuncil, Xin Nie, Amandeep Kaur, Bradley L Reuhs, Eric C Martens, Bruce R Hamaker

**Affiliations:** Whistler Center for Carbohydrate Research, Department of Food Science, 745 Agriculture Mall Drive, Purdue University, West Lafayette, IN 47907, United States; Present address: Nestlé Health Science, Shanghai, P.R. China; Department of Microbiology and Immunology, Medical Sciences Research Building II, 1150 W Medical Center Dr., University of Michigan Medical School, Ann Arbor, MI 48109, United States; Whistler Center for Carbohydrate Research, Department of Food Science, 745 Agriculture Mall Drive, Purdue University, West Lafayette, IN 47907, United States; Food Engineering Department, Yeni Meram Boulevard Kasım Halife Street, Necmettin Erbakan University, Konya 42090, Turkey; Whistler Center for Carbohydrate Research, Department of Food Science, 745 Agriculture Mall Drive, Purdue University, West Lafayette, IN 47907, United States; Whistler Center for Carbohydrate Research, Department of Food Science, 745 Agriculture Mall Drive, Purdue University, West Lafayette, IN 47907, United States; Whistler Center for Carbohydrate Research, Department of Food Science, 745 Agriculture Mall Drive, Purdue University, West Lafayette, IN 47907, United States; Department of Microbiology and Immunology, Medical Sciences Research Building II, 1150 W Medical Center Dr., University of Michigan Medical School, Ann Arbor, MI 48109, United States; Whistler Center for Carbohydrate Research, Department of Food Science, 745 Agriculture Mall Drive, Purdue University, West Lafayette, IN 47907, United States

**Keywords:** glycans, dietary fiber, arabinoxylan, gut, microbiome, Bacteroides

## Abstract

Structurally complex corn bran arabinoxylan (CAX) was used as a model glycan to investigate gut bacteria growth and competition on different AX-based fine structures. Nine hydrolyzate segments of the CAX polymer varying in chemical structure (sugars and linkages), CAX, five less complex non-corn arabinoxylans, and xylose and glucose were ranked from structurally complex to simple. The substrate panel promoted different overall growth and rates of growth of eight *Bacteroides* xylan-degrading strains. For example, *Bacteroides cellulosilyticus* DSM 14838 (*Bacteroides cellulosilyticus*) grew well on an array of complex and simple structures, while *Bacteroides ovatus* 3-1-23 grew well only on the simple structures. In a competition experiment, *B. cellulosilyticus* growth was favored over *B. ovatus* on the complex AX-based structure. On the other hand, on the simple structure, *B. ovatus* strongly outcompeted *B. cellulosilyticus*, which was eliminated from the competitive environment by Day 11. This adaptation to fine structure and resulting competition dynamics indicate that dietary fiber chemical structures, whether complex or simple, favor certain gut bacteria. Overall, this work supports a concept that fiber degraders diversify their competitive abilities to access substrates across the spectrum of heterogeneity of fine structural features of dietary fibers.

Colonic microorganisms mainly ferment undigested dietary carbohydrates for energy support. The microbial diversity coexisting in the human colon indicates bacteria possess exclusive abilities to utilize diverse substrate structures, ensuring survival and proliferation in such a competitive environment [1–6]. While fiber complexity (sugar composition, linkage types, and branching degree) can create growth niches for bacteria uniquely equipped with matching complex degradation systems, it is less evident whether such niches are also created with simpler fiber structures.

We recently reported that corn bran arabinoxylan (CAX) has a complex but homogenous (monodisperse) structure with highly branched complex subunits flanked by more simple, unsubstituted regions [7]. These features made CAX a good model fiber for investigating, in the current study, the relationship between fiber structure complexity and growth of xylan-degrading *Bacteroides spp*. We hypothesized that gut bacteria fill niches in which they best grow and compete based on both high and low structural complexity, and that this can occur even within one polysaccharide (i.e. CAX).

A range of AX structures varying in branching degree and branch complexity were created using sequential enzymatic treatments from a CAX polymer (Fig. S1a, see online supplementary material for a colour version of this figure**,** Supplementary Materials and Methods). Structural data were applied to simple algorithms set up to give scores related to structural complexity, and nine CAX hydrolyzates were further selected for experiments based on gradated differences in “degree of branching,” and “branch complexity” (Fig. S1b–d, see online supplementary material for a colour version of this figure; Tables S1–S3). Other simpler AX-based structures were obtained (Supplementary Materials and Methods) yielding a panel of 15 AX-based structures that were used for bacterial growth experiments.

AX-based structures were ranked high-to-low in order of complexity with the 10 CAX-based substrates described above making up the more complex structures followed, in rank order of structure complexity (high-to-low), sorghum and rice AX (SAX, RAX), debranched sorghum AX (SDB), wheat endosperm AX (WAX), and oxalic acid debranched corn AX (CAH). Xylose and glucose were placed at the bottom of the ranking. The high-to-low structural complexity ranking of AX-based substrates provided a kind of “fingerprint” mapping for the growth of each *Bacteroides* strain tested (Fig. S1d, see online supplementary material for a colour version of this figure).

**Figure 1:**
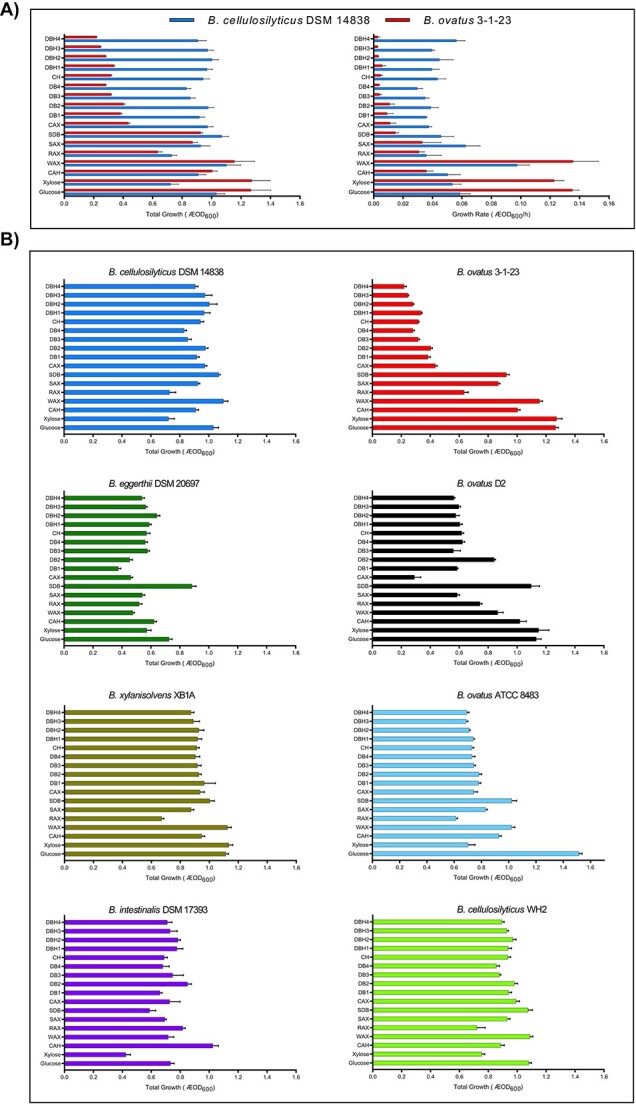
*Bacteroides spp.* growth patterns on cereal-based arabinoxylans ranked by level of structural complexity; (A) comparison of total growth (ΔOD_600_) and growth rate (ΔOD_600_/h) of *B. ovatus* 3-1-23 and *B. cellulosilyticus* DSM 14838 on a matrix of substrates with different structural complexity (high to low complexity, top to bottom); (B) total growth (ΔOD_600_) *B. ovatus* 3-1-23, *B. cellulosilyticus* DSM 14838, *B. ovatus* D2, *B. intestinalis* DSM 17393, *B. cellulosilyticus* WH2, *B. xylanisolvens* XB1A, *B. ovatus ATCC* 8483, and *B. eggerthii* DSM 20697 on a matrix of substrates with different structural complexity (high to low complexity, top to bottom), and all values are presented as the mean ± SEM; substrate abbreviations: CAH = corn bran arabinoxylan acid hydrolyzate, WAX = commercial water-soluble wheat AX, RAX = alkali-extracted rice bran AX, SAX = alkali-extracted sorghum bran AX, SDB = SAX debranched serially with AXH-d3 and AXH-m, CAX = alkali-extracted bran corn AX, DB1 = debranched CAX with AXH-m, DB2 = debranched CAX with AXH-d3, DB3 = debranched CAX serially with AXH-m and AXH-d3, DB4 = debranched CAX serially with AXH-d3 and AXH-m, CH = CAX hydrolyzate, DBH1 = DB1 endoxylanase hydrolyzate, DBH2 = DB2 endoxylanase hydrolyzate, DBH3 = DB3 endoxylanase hydrolyzate, and DBGH4 = DB4 endoxylanase hydrolyzate.

**Figure 2:**
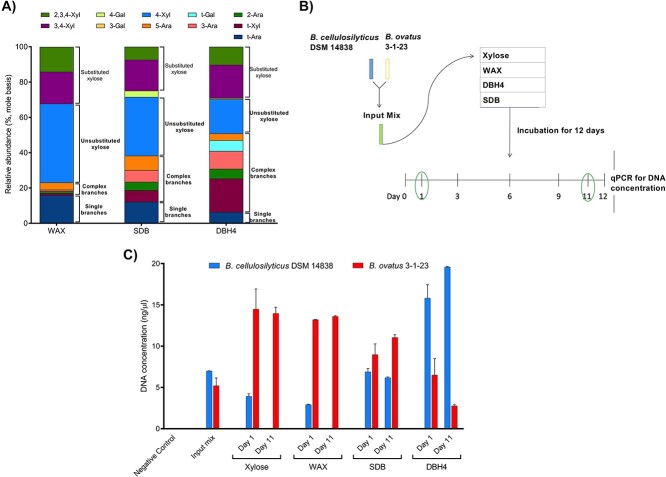
Competitive behavior of *Bacteroides* strains on xylose and arabinoxylans of increasing structural complexity; (A) structural features of WAX, SDB, and DBH indicated by their linkage profiles; from WAX to SDB to DBH, the structural complexity of the three samples progressively increases, and (B) schematic procedure for the mixed strains growth experiment, and the culture of two strains, *B. cellulosilyticus* DSM 14838 and *B. ovatus* 3-1-23, were mixed in a 1:1 ratio (v/v) and then incubated with four substrates: xylose, WAX, SDB, and DBH4; samples were harvested at Days 1 and 11; DNA concentration of each strain was determined, and negative controls were included by using the specific probe for one strain to detect the other one; (C) competition of *B. cellulosilyticus* DSM 14838 and *B. ovatus* 3-1-23 on xylose, WAX, SDB, and DBH4; the zero values of negative controls confirmed the specificity of the designed probes for each strain; values are presented as the mean ± SEM; substrate abbreviations: WAX = commercial water-soluble wheat AX, SDB = SAX debranched serially by AXH-d3 and AXH-m (SDB), and DBH4 = debranched CAX serially debranched with AXH-d3 and AXH-m (DB4), and then endoxylanase-hydrolyzed; negative controls showed zero DNA concentration, an indicator of growth, and the initial DNA concentration of each strain before incubation with the four different substrates is shown as “input mixture” in Fig. 2B.

Eight xylan-degrading *Bacteroides* common in the human gut [8, 9] were assessed for growth in pure cultures with the selected AX-based substrates. Each *Bacteroides* strain showed its own unique signature of growth on the array of ranked AX-based substrates (Figs 1, S2, see online supplementary material for a colour version of this figure). *Bacteroides cellulosilyticus* (14838 and WH2) had the highest total growth on most AXs, demonstrating a broad substrate utilization capacity on complex and simple structures. In agreement, growth curves on corn arabinoxylan hydrolyzate (CH) substrate showed a phase shift during *B. cellulosilyticus* growth, opposed to the other strains, which was followed by further growth (Fig. S3, see online supplementary material for a colour version of this figure), suggesting that *B. cellulosilyticus* can utilize the more complex AX parts. On the other hand, *Bacteroides ovatus* 3-1-23 showed distinctly lower total growth on complex CAX hydrolyzates, CAX, and RAX (cell density at ΔOD_600_ below 0.4 and mostly around 0.3). Its total growth on simpler substrates (CAH, WAX, SAX, SDB, xylose, glucose) was high, mostly around 1.0 (Fig. 1B). The other two *B. ovatus* strains (8483 and D2) showed somewhat better utilization of complex AX structures, indicating fiber utilization abilities vary at the strain level. Both *Bacteroides eggerthii* and *Bacteroides intestinalis* had only fair growth (0.4–0.8 ΔOD_600_) on the substrate panel, while *Bacteroides xylanisolvens* showed fairly good growth (above 0.8 ΔOD_600_) on almost all substrates (except RAX) (Fig. 1B). While all strains were able to grow on most substrates, the diversity of growth response suggests that each *Bacteroides* strain has evolved to possess different xylan utilization systems encoding binding proteins and enzymes specific to different structural features, which ensures their survival in the gut highly competitive environment.

*Bacteroides* growth profiles provided the basis to select two strains differing most in growth on complex and simple structures: *B. cellulosilyticus* 14838 for the former and *B. ovatus* 3-1-23 for the latter (Fig. 1). These *Bacteroides* strains were co-cultured on three arabinoxylan-based structures (selection criteria described in Supplementary Materials and Methods) of differing structural complexity (DBH4 > SDB > WAX > xylose) (Fig. 2A and B).

When grown on the structurally complex DBH4, *B. cellulosilyticus* DSM 14838 growth was favored over *B. ovatus* 3-1-23; with the latter showing diminished growth from Days 1 to 11, though with retained viability (Fig. 2C). For SDB, the medium structural complexity AX, both strains showed a competitive ability to grow on Day 11. On the other hand, while both strains grew well on xylose and WAX when pure-cultured, in co-culturing experiments, *B. cellulosilyticus* 14838 had little ability to compete in these simpler substrates and was rapidly extinguished by *B. ovatus* 3-1-23. Notably, despite the high competitiveness of *B. cellulosilyticus* 14838 for complex substrates, *B. ovatus* 3-1-23 still was able to survive in co-culture experiments, likely due to its strong competitiveness for simple branched parts of SDB and DBH4.

While *B. cellulosilyticus* 14838 exhibited growth abilities across a wide range of fiber structures, as indicated by our pure-culture experiments and supported by others [10], it was less efficient to utilize simple structures in competitive environments. *Bacteroides ovatus* 3-1-23, though less capable of degrading a broad array of AXs structures, outgrew and outcompeted *B. cellulosilyticus* 14838 in simple structures. *Bacteroides ovatus* and *B. cellulosilyticus* are important commensal colonic bacteria with major roles in xylan degradation [10, 11] and were, thus, used as model organisms to understand how niche specialization to fiber structure can confer growth advantage. Notably, the *in vitro* competition model using selected strains of these two species, as presented here, does not capture the full intricacy of the gut microbiota dynamics. Besides its significant diversity, the large intestine also receives an influx of numerous other glycans. These may be prioritized based on competitive pressures, facilitating the stable coexistence of diverse microbial populations [6]. Thus, further examination is warranted to demonstrate the relevance of such fine fiber structure niche specializations to the human gut microbiome.

Overall, whether complex or simple, discrete chemical structures align with bacterial strains that allow them to compete and grow. Such niches for bacterial growth that occur even within the same polymer (e.g. CAX) have the potential to promote multiple bacterial taxa with a single fiber type, supporting previous observations of higher diversity after fermentation of structurally complex carbohydrates compared with simpler ones [12]. Interestingly, even in the case of simple chemical structures, where all degraders would have nearly equal access to the contained sugars with fewer sugar types and linkages, bacteria still have abilities to outcompete others. This reveals bacterial niche specialization and alignment that can be observed even among simple fiber structures.

## Supplementary Material

Xu_et_al_ISME_Supplement_Revision_No_Markups_ycae037

## Data Availability

The data that support the findings of this study are available in Open Science Framework, DOI 10.17605/OSF.IO/2R3UA.
