# Supplementary material for: When simplicity triumphs: niche specialization of gut bacteria exists even for simple fiber structures
Source: ISME Commun. 2024 Apr 11;4(1):ycae037. doi: 10.1093/ismeco/ycae037 (PMC11032216; doi:10.1093/ismeco/ycae037)
Supplement: Xu_et_al_ISME_Supplement_Revision_No_Markups_ycae037 [file xu_et_al_isme_supplement_revision_no_markups_ycae037.docx]

Supplementary Information for

**When simplicity triumphs: Niche specialization of gut bacteria exists even for simple fiber structures**

Haidi Xu^1,4^, Nicholas A. Pudlo^2^, Thaisa M. Cantu-Jungles^1^, Yunus E. Tuncil^3^, Xin Nie^1^, Amandeep Kaur^1^, Bradley L. Reuhs^1^, Eric C. Martens^2,*^, and Bruce R. Hamaker^1,*^

^1^ Whistler Center for Carbohydrate Research, Department of Food Science, Purdue University, West Lafayette, IN 47907, USA

^2^ Department of Microbiology and Immunology, University of Michigan Medical School, Ann Arbor, MI 48109, USA

^3^ Food Engineering Department, Necmettin Erbakan University, Konya, 42090, Turkey

^4^ Present address: Nestlé Health Science, Shanghai, P.R. China

^*^ Corresponding authors:

Eric C. Martens ([emartens@umich.edu](mailto:emartens@umich.edu)), Department of Microbiology and Immunology, University of Michigan Medical School, Ann Arbor, MI 48109, USA, 1-734-645-2775

and

Bruce R. Hamaker ([hamakerb@purdue.edu](mailto:hamakerb@purdue.edu)), Whistler Center for Carbohydrate Research and Department of Food Science, Purdue University, West Lafayette, IN 47907, USA; hamakerb@purdue.edu; 1-765-494-5668

List of contents:

1. Supplementary Materials and Methods
2. Supplementary Tables
3. Supplementary Figures
4. References

**1. Materials and methods**

Corn AXs structural features and fermentability experiments

*Materials*

Dry-milled corn bran (particle size ﹤50 μm) was a gift from Bunge Milling (Danville, IL); rice and wheat brans were store-bought; and sorghum bran was obtained from decorticating locally grown sorghum from the Purdue Agronomy Farm. Multifect^®^ CX XL endoxylanase was a gift from Genencor International Inc. (Rochester, NY). Arabinoxylan arabinofuranohydrolase AXH-d3 and AXH-m were gifts from Novozymes North America Inc (Franklinton, NC) and detailed enzymes preparation was reported by [1]. AXH-d3 selectively hydrolyzes the (1→3)-α-L-arabinofuranosyl residues of doubly substituted xylopyranosyl residues while AXH-m catalyzes the removal of both (1→2) and (1→3)-α-L-arabinofuranosyl residues from singly substituted xylopyranosyl residues in AXs. Other enzymes and chemicals were purchased from Sigma-Aldrich Co. (St. Louis, MO).

*AX purification*

Alkali-extractable AXs were obtained from corn, rice, wheat and sorghum brans by the alkaline-hydrogen peroxide extraction method as previously described by Doner et al. [2] with modifications by Rose et al. [3]. Fractionation of the extracted AXs was performed by the method of Verwimp et al. [4], with minor modifications. In general, extracted AXs were solubilized in de-ionized water (5% w/v), pure ethanol was added to a final concentration of 40% and kept overnight at 4^o^C. Insoluble materials were removed by centrifugation (10,000 *g*, 30 min, 15 ºC). The concentration of ethanol was increased to 60%, kept overnight at 4^o^C and after centrifugation (10,000 *g*, 30 min, 15 ºC) the aqueous ethanol portions were discarded. The remaining precipitates were dried (45 ºC, ~ 36h), and grounded into a powder yielding fractions CAX (corn arabinoxylan), RAX (rice arabinoxylan), WAX (wheat arabinoxylan) and SAX (sorghum arabinoxylan).

*Preparation of hydrolyzate fractions*

A portion of CAX was hydrolyzed as previously described [4], with minor modifications. CAX was dissolved in sodium acetate buffer (25 mM, pH ~ 5.0) at 2% concentration (w/v%), and 15 ml of Multifect^®^ CX XL endoxylanase (445 XAU/ml) was added. The mixture was incubated with shaking (60 ºC, 24 h) and further boiled for enzyme inactivation. Absolute ethanol (4 vol.) was added to precipitate the corn hydrolyzate (CH), which was recovered through centrifugation (10,000 *g*, 30 min, 15 ºC).

CAX and CH were sequentially debranched by AXH-d3 and/or AXH-m enzymes in distinct order patterns as indicated in Fig. S1a (AXH-d3 only, AXH-m only, AXH-m followed by AXH-d3 or AXH-d3 followed by AXH-m). Debranching treatments were performed as previously described [1], with some modifications. Briefly, samples were dissolved in sodium acetate buffer (25 mM, pH 6) at 20% (w/v) concentration. AXH-d3 or AXH-m enzymes were added (0.4% v/v) and incubated with constant stirring, at 40 ºC for 6 h (AXH-d3) or 12 h (AXH-m). Absolute ethanol (4 vol.) was added to precipitate the resultant hydrolyzates, which were recovered through centrifugation (10,000 *g*, 30 min, 15 ºC).

Finally, hydrolyzates resultant from CAX debranching (DB1 to DB4, Fig. S1a) were further hydrolyzed by endoxylanase as described above for CH yielding fractions DBH1 to DBH4 (Fig. S1a).

*Total carbohydrate content and monosaccharide composition*

Total carbohydrate was determined by the phenol-sulfuric acid method [5]. Total monosaccharide composition was determined by gas chromatography (GC) using the modified procedures described by Rose et al. [3] to a smaller sample size (50 mg). Reducing-end and monomeric monosaccharide compositions were evaluated according to Courtin et al.[6]. Alditol acetates in ethyl acetate were quantified by gas chromatography using a capillary column SP-2330 (Supelco, Bellefonte, PA) with the following conditions: injector volume, 2 μl; injector temperature, 240 ºC; detector temperature, 300 ºC; carrier gas (helium), velocity 1.9 meter/second; split ratio, 1:2; temperature program was 160 ºC for 6 min, then 4 ºC/min to 220 ºC for 4 min, then 3 ºC/min to 240 ºC for 5 min, and then 11 ºC/min to 255 ºC for 5 min.

*Glycosidic linkage profile analysis*

Linkage analysis was performed by the method of Carpita and Shea [7] with modifications: dried samples (3 mg) were dissolved in anhydrous DMSO (300 μl) and methylated. Methylated samples were hydrolyzed using 250 μl of 2 N trifluoroacetic acid (121 ºC for 1 h). The hydrolyzed samples were dissolved in 100 μl of 1M ammonium hydroxide, and 500 μl of DMSO containing 20 mg/ml of sodium borodeuteride. The mixture was incubated at 40 ºC for 90 min and 6-9 drops of glacial acetic acid were added and mixed to stop reduction reaction. 1-methylimidazole (100 μl) and acetic anhydride (500 μl) were added for acetylation. Partially methylated alditol acetates in acetone were quantified by GC-FID-MS (7890A-5975C MSD, Agilent Technologies, Inc., Santa Clara, CA, USA) using a SP-2330 capillary column with the following conditions: injector volume, 1 μl; injector temperature, 240 ºC; detector temperature, 300 ºC; carrier gas, helium: 1.9 meter/second; split ratio, 100:1; temperature program, 100 ºC for 2 min, 8 ºC/min to 240 ºC for 20 min.

*Obtention of other arabinoxylan structures*

Other 5 AX-based structures of lesser branch complexity had been obtained as follows: alkali-extracted sorghum and rice AX (SAX, RAX); sorghum AX sequentially debranched by AXH-d3 followed by AXH-m (SDB) in the same conditions described above for CAX and CH; commercial wheat endosperm water-soluble AX (WAX), and oxalic acid debranched corn AX (CAH).

Bacteroides growth experiments

*Microorganisms and media*

The following eight publicly available Bacteroides strains belonging to 5 different species were included in this study: *Bacteroides cellulosilyticus* DSM 14838, *Bacteroides cellulosilyticus* WH2, *Bacteroides ovatus* ATCC 8483, *Bacteroides ovatus* 3-1-23, *Bacteroides ovatus* D2, *Bacteroides eggerthii* DSM 20697, *Bacteroides intestinalis* DSM 17393, *Bacteroides xylanisolvens* XB1A. Except *B. cellulolyticus* WH2, a draft genome sequence available for the other 7 strains has been completed.

Growth experiments were performed in Bacteroides minimal medium (MM) as described previously [8], containing 100 mM KH_2_PO_4_ (pH 7.2), 15 mM NaCl, 8.5 mM (NH_4_)_2_SO_4_, 4 mM L-cysteine, 1.9 µM hematin/200 µM L-histidine, 100 μM MgCl_2_, 1.4 µM FeSO_4_•7H_2_O, 50 µM CaCl_2_, 1 µg ml^-1^ vitamin K_3_, and 5 ng ml^-1^ vitamin B_12_. All carbon sources were added to MM at a final concentration 0.5% (w/v) and were sterilized by autoclaving in distilled water at 1% (w/v). Medium was reduced by adding L-cysteine just before use and immediately placed into anaerobic chamber (Coy Manufacturing, Grass Lake, MI).

*Substrates*

CAX, all CAX fractions obtained from enzymatic (CH, DB1 to DB4, DBH1 to DBH4 and HDB1 to HDB4, as described in Fig. 3A) and acid (CAH) hydrolysis, SAX and its hydrolysate fraction SDB, WAX, RAX, and monosaccharides glucose and xylose were used for pure culture growth experiments.

*Pure strain growth experiment*

Diluted bacterial inoculum (100 μl, 4%) was inoculated into plate wells pre-loaded with 100 μl of each substrate described above (10 mg/ml) individually. The plates were incubated in the anaerobic chamber for 40 h at 37 ºC and bacterial growth was monitored by reading the turbidity at OD_600_.

An initial experiment including six *Bacteroides* strains (identified in **Fig. S1**) grown separately on CH was performed. Then an expanded growth experiment including the eight strains listed at the top of this section and 15 AX-based structures (described above in “Substrates”) and xylose and glucose was conducted to provide a more comprehensive understanding about the substrate preference pattern of each strain and to enable the selection of strains and substrates efficiently for the following competition study. Total growth and overall growth rate were calculated based on the bacterial growth curves.

*Arabinoxylan structures selection for mixed strains growth experiment*

Three of the above-described AX substrates and xylose were selected for the competition study, using the following selection criteria. Two AX substrates (DBH4 and SDB) were chosen due to their high total growth and good growth rate for *B. cellulosilyticus* DSM 14830, but different structural complexity levels (high and moderate, respectively). DBH4 was the most structurally complex AX substrate in the panel (Fig. S1b-d), and showed both higher total growth and growth rate for *B. cellulosilyticus* DSM 14830 compared to *B. Ovatus* (Fig 1, Fig. S2) ; and SDB, a moderately complex AX from sorghum, showed somewhat higher total growth for *B. cell* than *B. ovatus*, but substantially higher growth rate for *B. cell*. In contrast, WAX and xylose were selected to favor *B. ovatus*. WAX, a lightly branched AX with a high number of unsubstituted regions (Fig. S1b-d), had similar total growth for the two strains, but growth rate was notably higher for *B. ovatus* (Fig 1, Fig. S2). For xylose, *B. ovatus* had much higher total growth and growth rate compared to *B. cellulosilyticus* (Fig 1, Fig. S2). *B. cellulosilyticus* DSM 14830 and *B. ovatus* 3-1-23 were co-cultured on the substrates (by structural complexity, DBH4 > SDB > WAX > xylose).

*Mixed strains growth experiment*

The mixture of the two strains, *Bacteroides cellulosilyticus* DSM 14838 and *Bacteroides ovatus* 3-1-23, in a 1:1 ratio (v/v), was incubated with four substrates xylose, WAX, SDB and DBH4 as described above. Two μl of each culture was inoculated into another 200 μl of fresh substrate solution (10 mg/ml) plus minimum medium every day. Incubation was conducted for eleven days and samples were harvested at Day 1 and Day 11. DNA concentration of each strain was determined with specifically designed probes using a DNeasy Blood and Tissue Kit (Qiagen, Valencia, CA) according to the manufacturer’s instructions.

The primers used for *Bacteroides cellulosilyticus* 14838, targeting the species' 16S rRNA gene, and *Bacteroides ovatus* 3-1-23, targeting the TonB-dependent receptor SusC homolog with locus tag HMPREF9010_04166, had the following sequences:

|  | *Bacteroides cellulosilyticus 14838* | *Bacteroides ovatus 3-1-23* |
| --- | --- | --- |
| Forward | ataccaccccgcccactga | gggcgcagacatcagcagtt |
| Reverse | tgacgccaccacggaaaga | aaggcaaaatcataataatcccagtca |

Data availability

The data that support the findings of this study are available in GitHub, <https://github.com/ThaisaJungles/Haidi_Xu>.

**2. Supplementary Tables**

Table S1. General information for corn arabinoxylan and its nine enzymatic products.

| Sample Name | Treatment(s) | Abbrev. | Yield %(w/w) | Average M*w* (Da) |
| --- | --- | --- | --- | --- |
| Corn AX | Alkali-extracted AX from purified corn bran | CAX | 40.0 | 1.9×106 |
| Debranched corn AX1 | CAX debranched by AXH-m | DB1 | 69.5 | 8.2×105 |
| Debranched corn AX2 | CAX debranched by AXH-d3 | DB2 | 76.9 | 7.8×105 |
| Debranched corn AX3 | CAX debranched by AXH-m then AXH-d3 | DB3 | 62.8 | 6.8×104 |
| Debranched corn AX4 | CAX debranched by AXH-d3 then AXH-m | DB4 | 47.3 | 7.7×104 |
| Corn AX hydrolyzate | CAX hydrolyzed by endoxylanase | CH | 51.6 | 3.4×104 |
| Debranched corn AX1 hydrolyzate | DB1 hydrolyzed by endoxylanase | DBH1 | 67.5 | 4.8×104 |
| Debranched corn AX2 hydrolyzate | DB2 hydrolyzed by endoxylanase | DBH2 | 50.3 | 2.1×104 |
| Debranched corn AX3 hydrolyzate | DB3 hydrolyzed by endoxylanase | DBH3 | 59.1 | 1.9×104 |
| Debranched corn AX4 hydrolyzate | DB4 hydrolyzed by endoxylanase | DBH4 | 66.4 | 1.7×104 |

Table S2. Neutral sugar composition for CAX and its nine enzymatic products.

|  | Neutral Sugar Composition %(w/w)^a^ | | | A/X ratio^b^ |
| --- | --- | --- | --- | --- |
|  | Ara | Xyl | Gal |  |
| CAX | 20.3± 1.0 | 39.8± 2.5 | 6.4± 0.3 | 0.51 |
| DB1 | 18.9 ± 0.1 | 42.0 ± 0.6 | 6.8 ± 0.0 | 0.45 |
| DB2 | 20.7 ± 0.6 | 44.7 ± 1.2 | 7.4 ± 0.2 | 0.46 |
| DB3 | 17.0 ± 0.4 | 47.1 ± 0.8 | 8.8 ± 0.4 | 0.36 |
| DB4 | 16.2 ± 0.4 | 45.1 ±0.9 | 9.0 ± 0.3 | 0.36 |
| CH | 20.1± 1.0 | 44.5± 2.6 | 8.5± 0.2 | 0.46 |
| DBH1 | 18.5 ± 0.7 | 43.5 ± 1.6 | 7.8 ± 0.4 | 0.43 |
| DBH2 | 17.5 ± 1.1 | 47.8 ± 4.9 | 8.9 ± 0.2 | 0.37 |
| DBH3 | 17.1 ± 1.0 | 50.2 ± 2.9 | 10.0 ± 0.4 | 0.34 |
| DBH4 | 17.5 ± 0.7 | 52.9± 1.8 | 10.8 ± 0.8 | 0.32 |
| CAX | 20.3± 1.0 | 39.8± 2.5 | 6.4± 0.3 | 0.51 |
| DB1 | 18.9 ± 0.1 | 42.0 ± 0.6 | 6.8 ± 0.0 | 0.45 |
| DB2 | 20.7 ± 0.6 | 44.7 ± 1.2 | 7.4 ± 0.2 | 0.46 |
| DB3 | 17.0 ± 0.4 | 47.1 ± 0.8 | 8.8 ± 0.4 | 0.36 |
| DB4 | 16.2 ± 0.4 | 45.1 ±0.9 | 9.0 ± 0.3 | 0.36 |

^a^ Mean ± standard error; a negligible amount of glucose content was also detected, 1.2% for CAX and zero or no more than 0.3% for the enzymatic products.

^b^ Calculated by the content of Ara divided by that of Xyl.

Abbreviations: Ara = arabinose, Xyl = xylose, Gal = galactose , A/X ratio = the ratio of arabinose to xylose, CAX = corn alkali-extracted arabinoxylan fractionated by 40-60% ethanol, CH = Endoxylanase-hydrolyzate of CAX, DB1 = CAX debranched by removing terminal arabinosyl moieties from 2- or 3-mono-substitution, DB2 = CAX debranched by removing terminal arabinosyl moieties from 3-O position of 2,3-disubstitution, DB3 = CAX debranched by removing terminal arabinosyl moieties from 2- or 3-mono-substitution first and then from 3-O position of 2,3-disubstitution, DB4 = CAX debranched by removing terminal arabinosyl moieties from 3-O position of 2,3-disubstitution first and then from 2- or 3-mono-substitution, DBH1 = Endoxylanase-hydrolyzate of DB1, DBH2 = Endoxylanase-hydrolyzate of DB2, DBH3 = Endoxylanase-hydrolyzate of DB3, DBH4 = Endoxylanase hydrolyzate of DB4.

|  | t- Ara*f* | t-Xyl*p* | t-Gal*p* | 2-Ara*f* | 3-Ara*f* | 5-Ara*f* | 4-Xyl*p* | 3,4- or 2,5-Xyl*p* | 2,3,4-Xyl*p* |
| --- | --- | --- | --- | --- | --- | --- | --- | --- | --- |
| CAX | 15.8 ± 0.4 | 14.9 ±2.4 | 3.4 ±0.8 | 4.2 ± 0.2 | 6.5 ±0.8 | 2.5 ±1.3 | 12.1 ±0.1 | 24.9 ±1.9 | 14.2 ±2.3 |
| DB1 | 13.5 ± 0.9 | 15.5 ±0.6 | 3.4 ±0.4 | 4.4 ± 0.1 | 7.6 ±0.7 | 2.6 ±0.2 | 15.7 ±0.2 | 20.8 ±0.8 | 15.5 ±0.5 |
| DB2 | 13.8 ± 1.1 | 13.1 ±1.3 | 3.5 ±0.6 | 4.5 ± 0.3 | 7.9 ±0.2 | 3.1 ±0.8 | 12.0 ±0.4 | 30.2 ±1.0 | 10.2 ±1.1 |
| DB3 | 8.9 ± 0.6 | 15.9 ±0.5 | 4.2 ±0.1 | 4.3 ± 0.2 | 8.3 ±0.0 | 3.2 ±0.1 | 18.4 ±0.4 | 24.4 ±1.4 | 10.8 ±0.4 |
| DB4 | 8.4 ± 0.1 | 16.6 ±0.5 | 5.1 ±0.1 | 4.6 ± 0.1 | 8.8 ±0.4 | 3.3 ±0.0 | 18.7 ±0.1 | 22.5 ±0.2 | 10.5 ±0.5 |
| CH | 13.4 ± 0.7 | 17.7 ±0.5 | 5.2 ±0.1 | 4.5 ± 0.0 | 8.5 ±0.0 | 3.3 ±0.1 | 14.6 ±0.3 | 18.2 ±1.6 | 13.4 ±0.3 |
| DBH1 | 12.7 ± 0.2 | 17.3 ±0.7 | 4.3 ±0.1 | 4.8 ± 0.1 | 8.5 ±0.1 | 3.0 ±1.1 | 14.2 ±0.2 | 19.5 ±0.4 | 14.6 ±0.4 |
| DBH2 | 9.7 ± 0.3 | 19.6 ±1.2 | 5.9 ±0.7 | 5.2 ± 0.1 | 8.4 ±0.2 | 3.8 ±0.3 | 15.9 ±0.2 | 21.3 ±1.9 | 9.4 ±1.0 |
| DBH3 | 7.8 ± 0.1 | 19.7 ±0.9 | 5.9 ±0.0 | 5.5 ± 0.1 | 9.1 ±0.1 | 3.5 ±0.2 | 16.7 ±0.1 | 20.6 ±0.2 | 10.0 ±0.7 |
| DBH4 | 6.4 ± 0.3 | 18.9 ±0.6 | 6.1 ±0.0 | 5.5 ± 0.4 | 10.0 ±0.3 | 3.9 ±0.1 | 19.4 ±0.4 | 18.7 ±0.3 | 10.2 ±0.5 |

Table S3. Linkage profiles of CAX and nine enzymatic products (mol%).

Results expressed as mean ± standard error, based on samples methylation data

Abbreviations: CAX = corn alkali-extracted arabinoxylan fractionated by 40-60% ethanol, CH = Endoxylanase-hydrolyzate of CAX, DB1 = CAX debranched by removing terminal arabinosyl moieties from 2- or 3-mono-substitution, DB2 = CAX debranched by removing terminal arabinosyl moieties from 3-O position of 2,3-disubstitution, DB3 = CAX debranched by removing terminal arabinosyl moieties from 2- or 3-mono-substitution first and then from 3-O position of 2,3-disubstitution, DB4 = CAX debranched by removing terminal arabinosyl moieties from 3-O position of 2,3-disubstitution first and then from 2- or 3-mono-substitution, DBH1 = Endoxylanase-hydrolyzate of DB1, DBH2 = Endoxylanase-hydrolyzate of DB2, DBH3 = Endoxylanase-hydrolyzate of DB3, DBH4 = Endoxylanase hydrolyzate of DB4.

**3. Supplementary Figures**


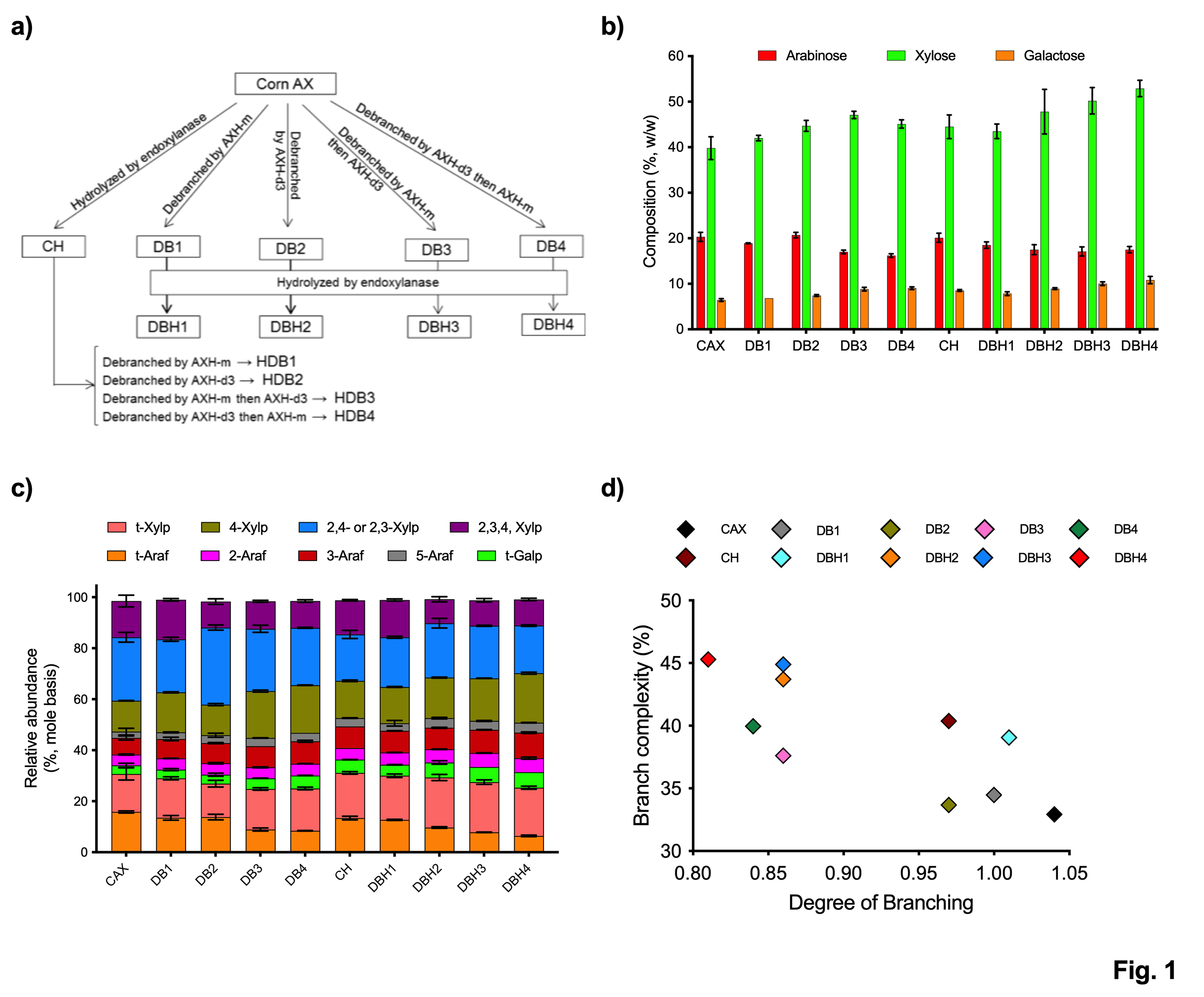


**Figure S1. Fractionation of corn bran arabinoxylan hydrolysates varying in structural complexity. a)** Enzymatic treatment procedure of CAX to generate fragments of differing structural complexity. **b)** Changing trend of the monosaccharide composition of CAX and its nine enzymatic products. Values are presented as the mean ± SEM. **c)** Changing trend of the content of glycosidic linkages of CAX and its nine enzymatic products based on partial methylation analysis. Values are presented as the mean ± SEM. **d)** Branching patterns of CAX and its nine enzymatic products in respect to branch complexity and degree of branching. Branch complexity = Sum (terminal Xyl + 2-Ara + 3-Ara + terminal Gal + 5-Ara + 3-Gal + 4-Gal); degree of branching = Sum (mono- + [di-*2])-substituted Xyl / total Xyl. Abbreviations: CAX = corn bran alkali-extracted arabinoxylan fractionated by 40-60% ethanol, CH = endoxylanase-hydrolyzate of CAX, DB1 = CAX debranched by the removal of terminal arabinosyl moieties from 2- or 3-mono-substitution, DB2 = CAX debranched by the removal of terminal arabinosyl moieties from 3-*O* position of 2,3-disubstitution, DB3 = CAX debranched by the removal of terminal arabinosyl moieties from 2- or 3-mono-substitution first and then from 3-*O* position of 2,3-disubstitution, DB4 = CAX debranched by the removal of terminal arabinosyl moieties from 3-*O* position of 2,3-disubstitution first and then from 2- or 3-mono-substitution, DBH1 = endoxylanase hydrolyzate of DB1, DBH2 = endoxylanase hydrolyzate of DB2, DBH3 = endoxylanase hydrolyzate of DB3, DBH4 = endoxylanase hydrolyzate of DB4.

**
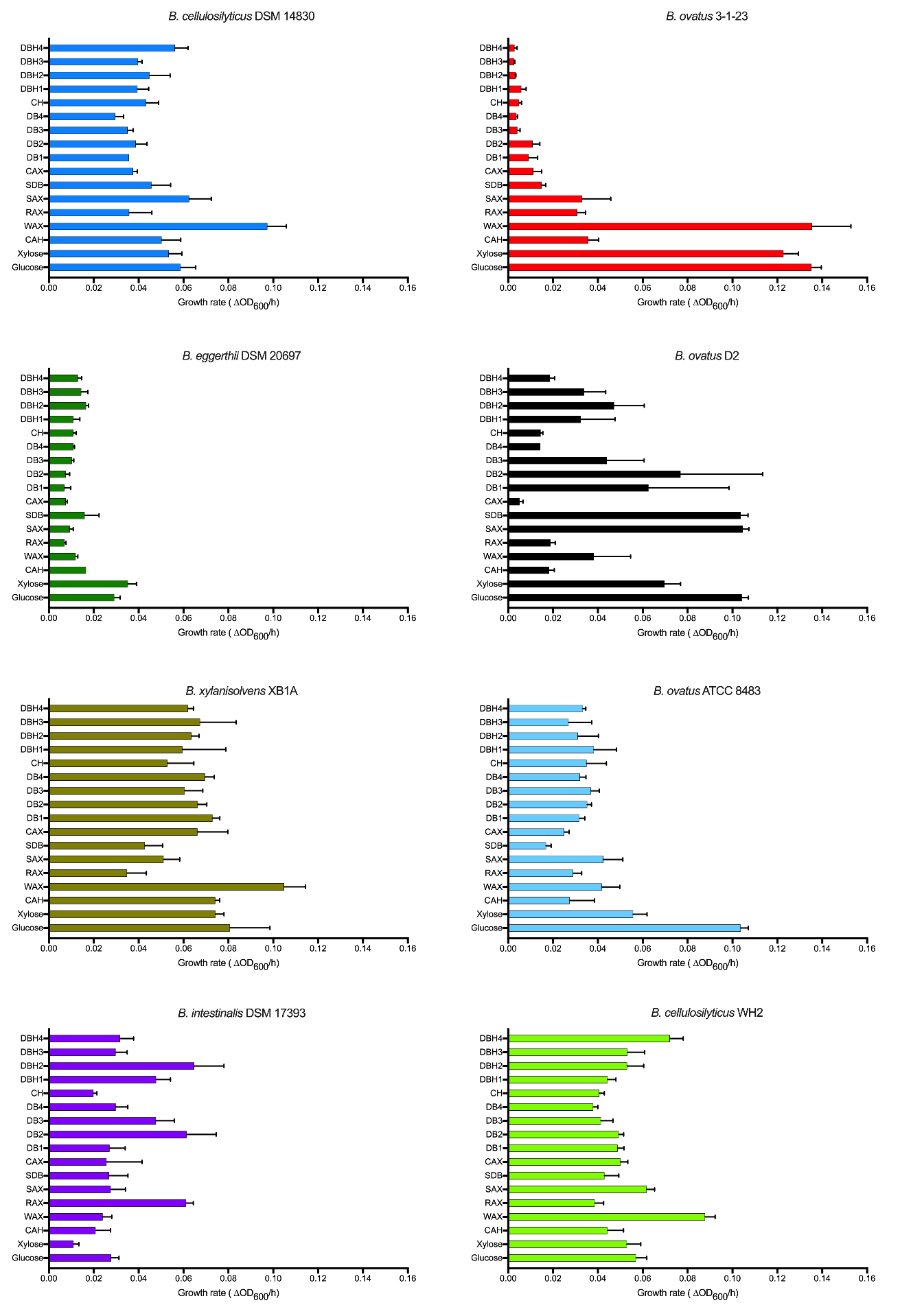
**

**Figure S2.** Overall growth rate (ΔOD600/ h) of *B. ovatus* 3-1-23, *B. cellulosyliticus* DSM 14830, *B. ovatus* D2, *B. intestinalis* DSM 17393, *B.* *cellulosyliticus* WH2, *B. xylanisolvens* XB1A, *B. ovatus ATCC* 8483 and *B. eggerthii* DSM 20697 on a matrix of substrates with different structural complexity (high to low complexity, top to bottom). Substrates abbreviations: corn acid hydrolyzates (CAH), commercial wheat AXs (WAX), alkali-extracted rice AXs (RAX), alkali-extracted sorghum AXs (SAX), SAX debranched subsequently by AXH-d3 and AXH-m (SDB), alkali-extracted corn AXs (CAX), debranched CAX by AXH-m (DB1), debranched CAX by AXH-d3 (DB2), debranched CAX subsequently by AXH-m and AXH-d3 (DB3), debranched CAX subsequently by AXH-d3 and AXH-m (DB4), CAX hydrolyzates (CH), DB1 hydrolyzates (DBH1), DB2 hydrolyzates (DBH2), DB3 hydrolyzates (DBH3), and DB4 hydrolyzates (DBH4).


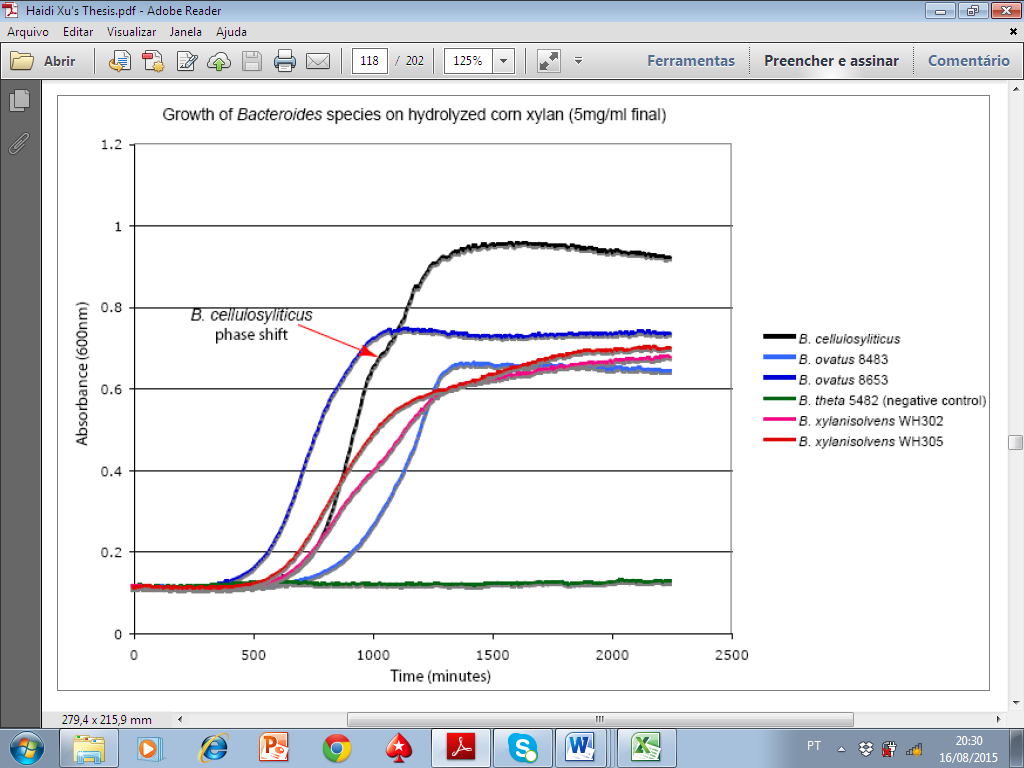


**Figure S3.** Bacterial growth curves of six strains from different *Bacteroides* species on the same corn arabinoxylan molecule. Different total growth and overall growth rate were observed for each strain. Phase shift occurred during the growth of *B. cellulosyliticus*, which was followed by a continuous growth, indicating a further utilization of the substrate. In contrast, the population increase of other strains stopped at a similar growth point when the phase shift occurred.

4. **References**

1. Sørensen HR, Jørgensen CT, Hansen CH, Jørgensen CI, Pedersen S, Meyer AS. A novel GH43 alpha-L-arabinofuranosidase from Humicola insolens: mode of action and synergy with GH51 alpha-L-arabinofuranosidases on wheat arabinoxylan. *Appl Microbiol Biotechnol* 2006; **73**: 850–861.

2. Doner LW, Hicks KB. Isolation of hemicellulose from corn fiber by alkaline hydrogen peroxide extraction. *Cereal Chem* 1997; **74**: 176–181.

3. Rose DJ, Patterson JA, Hamaker BR. Structural differences among alkali-soluble arabinoxylans from Maize (Zea mays), Rice (Oryza sativa), and Wheat (Triticum aestivum) brans influence human fecal fermentation profiles. *J Agric Food Chem* 2010; **58**: 493–499.

4. Verwimp T, Van Craeyveld V, Courtin CM, Delcour JA. Variability in the structure of rye flour alkali-extractable arabinoxylans. *J Agric Food Chem* 2007; **55**: 1985–1992.

5. Dubois M, Gilles KA, Hamilton JK, Rebers P, Smith F. Colorimetric method for determination of sugars and related substances. *Anal Chem* 1956; **28**: 350–356.

6. Courtin CM, Van Den Broeck H, Delcour JA. Determination of reducing end sugar residues in oligo- and polysaccharides by gas-liquid chromatography. *J Chromatogr A* 2000; **866**: 97–104.

7. Carpita NC, Shea EM. Linkage structure of carbohydrates by gas chromatography-mass spectrometry (GC-MS) of partially methylated alditol acetates. *Analysis of Carbohydrates by GLC and MS* 2021; 157–216.

8. Martens EC, Chiang HC, Gordon JI. Mucosal glycan foraging enhances fitness and transmission of a saccharolytic human gut bacterial symbiont. *Cell Host Microbe* 2008; **4**: 447–457.
